# Technology and instructor dimensions, e-learning satisfaction, and academic performance of distance students in Ghana

**DOI:** 10.1016/j.heliyon.2022.e09200

**Published:** 2022-03-31

**Authors:** Ahmed Bossman, Samuel Kwaku Agyei

**Affiliations:** Department of Finance, School of Business, University of Cape Coast, Cape Coast, Ghana

**Keywords:** Distance education system, Instructor factors, Technology anxiety, e-learning satisfaction, Learning outcomes

## Abstract

E-learning is soon expected to be widely used as a teaching and learning method in the mainstream for educational institutions. Given the relative preparedness of advanced economies, the conclusions about their implementation level with e-learning are incomparable with emerging countries. Emerging economies must, therefore, be aware of the issues to consider when formulating successful adoption or implementation strategies. However, empirical studies that bring forth these relevant factors are out of context. In a single framework, we model the structural relationships between the drivers of e-learning satisfaction and the performance of distance learning students in a frontier economy, Ghana. With 388 validated responses gathered from an online survey across the country between 29 May 2021 and 25 June 2021, we employ the Smart-PLS estimator to process and analyse the data. We explicate that the substantial drivers of e-learning satisfaction and performance among distance learning students include technology anxiety, instructor factors, course quality, technology quality, and ease of use. Our findings divulge that perceived learner satisfaction mediates the relationships between the drivers of satisfaction and learning outcomes of distance learning students in Ghana such that technology anxiety and instructor factors would not essentially enhance learner performance in the absence of e-learning satisfaction. Consequently, system quality, reflected by the information system success model must be supplemented by satisfaction, drawn from the expectation-confirmation theory, to fully explain the impact of efficient e-learning systems on learning outcomes. Not only does ease of use create satisfaction, but it also boosts performance. We, therefore, recommend institutions to develop regular training for both facilitators and students and also adopt user-friendly online platforms to aid patronage by learners.

## 1 Introduction

The rapid evolution and growth of e-learning, coupled with the several benefits it offers, has seen many colleges worldwide adopting e-learning systems. In the last decade, Bervell and Umar (2020) indicate that e-learning has been extensively adopted in higher educational levels. In Africa, a majority of colleges implement learning management systems (LMS) of several designs to supplement the traditional face-to-face arrangement. According to Adkins (2013), Africa's LMS adoption growth rate between 2011 and 2016 was projected to be 15% annually. In Palvia et al.'s (2018) recent projection, e-learning would become the mainstream by the year 2025. In Ghana, the adoption of e-learning was very low until the COVID-19 pandemic hit the country in March 2020, forcing institutions to adopt e-learning across both the distance and regular levels. A similar observation is reported by Sun et al. (2021), that the abrupt and unexpected state-wide shutdown of schools in Singapore, echoing the global scenario, has accelerated the use of technology in classrooms and pushed children to depend on home resources for reading. In situations where learners are left with no choice but to adapt to online learning, do they feel satisfied? What drives their satisfaction with the online system? Do they receive quality tuition? Are these courses of the quality desired? Do learners have the required technology to facilitate the online system?

It happens to be the case that the capacity of a reader to judge the credibility of material is critical to learning from reliable internet information (Kiili et al., 2021). Therefore, it is essential to know whether, in the course of online learning, learners can retrieve the right information concerning their course of study from the internet to afford them the quality and satisfaction they desire. Forson and Vuopala (2019) explain that Ghana seeks to widen the use of e-learning through the distance education system (DES). With the fast pace at which technology is advancing, an all-encompassing archetype of contemporary education is e-learning. From this backcloth, this study examines the key issues surrounding e-learning satisfaction and performance among distance learning students in Ghana. We seek to assess the future of e-learning in educational institutions by investigating: major factors that drive learner satisfaction for e-learning; how e-learning satisfaction influences the perceived performance or learning outcomes; whether perceived learner satisfaction mediates the relationship between the drivers of e-learning satisfaction and learning outcomes or performance. Should the channel through which the determining factors of e-learning satisfaction affect the performance of e-learners be of concern to educational policymakers in developing economies who aim to catch up with their counterparts in the advanced world?

After the first-hand experience, several users or initiators of e-learning put a stop to the system and little is yet discovered about why such a problem occurs. Tagoe (2012) opines that relative to advanced economies, emerging economies, particularly in Africa, are “crawling” (Palvia et al., 2018) in their adoption of e-learning, but in recent times, educational policymakers are on the verge of deploying proactive measures to catch up with their counterparts in the western world. The adoption of e-learning is influenced by the benefits the system comes with. Horn and Staker (2011) suggest that e-learning adoption becomes essential in the absence of a slot for traditional learning. In the words of Forson and Vuopala (2019), distance education is regarded as a major contributor to socio-economic development. Following this, it is expected that the policies that are rolled out to restructure or strengthen the DES are carefully designed to ensure improved contribution from this section of the education sector in developing economies. This has influenced the motivation for the extant literature on e-learning systems in Ghana.

In spite of the rising rate of e-learning adoption, Park (2009) notes that there exists a deficiency in its usage among both facilitators and students, and this elevates the worry about the success of LMS (Bervell & Umar, 2020). This has induced researchers in conducting studies on the e-learning system in Ghana. Empirical works on e-learning systems have focused on the integrated implementation (Jones, 2008), projections and encounters (Dadzie, 2009), the implementation (Serbe Marfo and Okine, 2011), policy guidelines (Osei et al., 2013), its growth (Kotoua et al., 2015), determinants of its adoption (Ansong, 2015), the readiness of students and institutions (Edumadze et al., 2017; Forson and Vuopala, 2019), and adoption determinants (Opoku et al., 2020).

E-learning is fundamentally a web-based programme that presents knowledge or information to learners readily on time regardless of time constraints or location proximity (Sun et al., 2008; Escobar Fandiño and Silva Velandia, 2020). Even though e-learning is known to possess a considerable number of merits over the usual “face-to-face” education (Kotoua et al., 2015), worries about the system include time, the intensity of manpower, and material capital (Bervell & Umar, 2020; Sun et al., 2008) required for the effective administration of the e-learning system. The costly and high propensity of failure of e-learning implementations discoursed by Arbaugh (2002), Bervell and Umar (2020), Edumadze et al. (2017), and Pérez-Pérez et al. (2020) merit consideration from administrators and designers of the e-learning system. Recent developments have called on scholars studying the determining factors of satisfaction with online education.

Escobar-Rodriguez and Monge-Lozano (2012) investigated the intention of learners toward the continuous use of the “Moodle” (an e-learning platform) under the tenets of the technology acceptance model (TAM) with 162 students. The study found support for the TAM, where ease of use (EU) and perceived usefulness (PU) had a significant influence on intention to use. In a cross-sectional investigation conducted by Ifinedo et al. (2018) in the Maritime section of Canada, 126 college students were surveyed to assess students' perception of usage, ease of use, and satisfaction with the Moodle in a unified learning atmosphere. The study found a positive impact of usability factors (PU and EU) on learner performance but no significant relationship was revealed for support from instructors or peers and performance. In the domain of online courses for colleges, Eom and Ashill (2016) examined the antecedents of learners’ satisfaction towards e-learning and expected learning performance. The respondents numbered 372 and comprised learners with at least one e-course completed at any university in the Midwestern US. The main independent variables studied were instructor factors, extrinsic and intrinsic learner motivation, learner self-regulation, and course structure, with perceived satisfaction and learner performance being the dependent variables. The results from the structural equation modelling (SEM) applied in the study revealed no significant effect of extrinsic learner motivation or learner self-regulation on learner performance. Dialogue between and among instructors and students, instructor factors and course structure were found to be significant drivers of both satisfaction and performance of e-learners. Learner intrinsic motivation was found a significant predictor of learner performance but not perceived satisfaction.

Under four broad dimensions titled “instructor, course, technology, and environment,” the perceived satisfaction of students in an e-learning framework was examined by Stefanovic et al. (2011) in Serbia. A survey instrument in the form of a questionnaire was administered to 143 participants and the data was processed using stepwise multiple regression analysis. The study found the significant drivers of learner satisfaction with e-learning to include promptness of instructor response, quality of technology, internet quality, diversified assessment, and interaction among users within the online environment. Contrary to Stefanovic et al.'s (2011) finding on the significance of interaction among users in the e-learning environment, Sun et al. (2008) had found that perceived interaction among learners was not a significant driver of learner satisfaction in an e-learning environment, even though operationalisation of the study variables were similar for both studies. With 295 respondents from Taiwan, Sun et al. (2008) performed stepwise multiple regression to determine key factors that contribute to learner satisfaction with e-learning systems under two extra dimensions (“design and learner”) as compared to that of Stefanovic et al. (2011). Different sample sizes and study locations could be attributable to the varying results.

Having applied the TAM and the information system success model (ISSM), Pérez-Pérez et al. (2020) evaluated the factors that determine learner satisfaction with the Moodle and learner performance among 155 college business learners of the University of Cantabria, a Spanish university. PLS was the technique for processing data and from the principles of the TAM and ISSM, the authors found the quality of information to be the principal determinant of learner satisfaction, which was found to be the main predictor of student performance in an e-learning environment at the higher education level. They indicate that the influence posed on learning performance by communicativeness is conditional to the educational framework – whether a blended mode or virtual studies or any applicable teaching context.

The determinants of Moodle effectiveness and satisfaction were studied by Damnjanovic et al. (2015) from the viewpoint of learners when they surveyed 255 e-learners in colleges within Bosnia, Herzegovina, Lithuania, and Serbia. PLS was the basis for estimating regression results and testing the study hypotheses, where out of 23 hypotheses, support was found for 16. The researchers employed, in line with their fundamental framework and in addition to perceived satisfaction, variables such as interactive intent for future use, communicativeness, structure, quality of information, perceived learning outcome, usefulness, and quality of the system. Contrary to the findings of Stefanovic et al. (2011) and Sun et al. (2008), Damnjanovic et al.'s (2015) study reported no significant influence of online “system and information” quality on perceived satisfaction, and also in opposite sides with Pérez-Pérez et al. (2020), Damnjanovic et al. (2015) rather found communicativeness as the most important predictor of learner performance in an e-learning environment.

In studying the predictors of instructor anxiety for LMS and its adoption in distance education in Ghana, Bervell and Umar (2020) employed the partial least squares (PLS) estimator to model the structural linkage between tutor anxiety on LMS and its implementation, with data from 267 facilitators located in the study centres across Ghana. They found peer stimulus, result expectancy and “use support” as the fundamental drivers of LMS-connected anxiety among tutors at the distance education level. Bervell and Umar (2020) revealed that peer stimulus and result expectancy were the key drivers of instructor anxiety for LMS and performance predictors of tutor anxiety for LMS, respectively. Opoku et al.’s (2020) study, although in Ghana, focused on lecturers only and focused on only one e-learning system, just like those from other technological frontier economies (see, e.g., Eom and Ashill, 2016; Ifinedo et al., 2018; Pérez-Pérez et al., 2020; etc.).

These studies have largely yielded differing results on the factors that drive the satisfaction of e-learners. Studies in developing economies, particularly Ghana, have yet to focus on the perceived satisfaction and performance of e-learning systems. It is instructive to note that, the level of advancement in technological frontier economies are incomparable to that of their emerging counterparts, especially in the African continent. Therefore, it stands to reason that, some findings from the extant literature focused on advanced economies may not apply to emerging countries that seem to be “crawling” in their adoption and/or implementation of e-learning (Palvia et al., 2018). Besides, there is the need to consider the effect of the satisfaction determining factors on the performance of users of e-learning systems. Furthermore, no study in Ghana has integrated the determining factors of e-learning satisfaction and perceived performance into a single framework subject to evaluation for corroboration and relationship testing. Thus, the channel through which the drivers of e-learning satisfaction affect the performance of e-learners remains a gap that needs to be given scholarly attention. As a result, we evaluate how the drivers of e-learning satisfaction influence learning outcomes and how their connections are explained by perceived satisfaction among distance learning students in Ghana. With the fast pace with which emerging economies are adopting and integrating e-learning in their education systems and the projected growth and wide usage of e-learning shortly, there may be little chance for “trial and error” approaches in developing countries owing to the high cost of implementation albeit high failure propensities; hence, we maintain that this study is timely.

Our contributions to the literature are fourfold. First, we focus on a developing economy, Ghana, to integrate the determining factors of e-learning satisfaction and perceived performance into a single framework subject to evaluation for corroboration and relationship testing. This provides novel and essential evidence for counterpart frontier economies that aim to implement e-learning systems. Second, investigations into the determining factors of satisfaction of e-learning systems and the direct effects they have on performance or learning outcomes as well as their indirect effects through perceived satisfaction on e-learning outcomes are essential to determine how future policies vis-à-vis the education system in developing economies would be handled by governments, institutional heads, as well as both tutors and learners. Third, the outcomes of an investigation like this should influence and enhance the digitisation and integration of educational systems across borders. The findings from our study would offer a guide for implementing an effective e-learning system across borders. Fourth, given the projection of Palvia et al. (2018) concerning the growth of e-learning and the fact that it may become mainstream by the year 2025, policymakers need to know what factors are essential to facilitate a smooth running of e-learning systems at the mainstream. This would enhance the efficiency of policy decisions and promote the growth and quality of education across the globe. The essence of empirical studies on the efficacy of e-learning systems concerning learning outcomes in this age puts our study in context.

We find from the empirical works reviewed that although some drivers of satisfaction with e-learning are contradicted in a few studies, a sizable number of the drivers are frequently found to be significant in predicting e-learners’ perceived satisfaction and performance. To test the significance of these variables within the Ghanaian context, we model, with verified observed variables, the constructs namely technology anxiety, course quality, technology quality, instructor factors, ease of use and expected usefulness together with perceived satisfaction and learner performance. The specific research questions that guide this study include:

1. What are the factors that drive the perceived satisfaction of e-learning among distance learning students in Ghana?
2. What is the impact of perceived e-learning satisfaction on the perceived performance of distance learning students?
3. Does perceived satisfaction of e-learning influence the relationships between the drivers of e-learning satisfaction and the perceived performance of learners?

This study provides relevant information to heads of institutions, policymakers, and relevant agencies in the context of distance education and online learning, by revealing which factors significantly predict the satisfaction of distance learning students and their expected performance in respect of e-learning especially in the case of developing economies, in which the previously crawling adoption rate is witnessing a boost owing to the COVID-19-induced constraints on traditional classroom arrangements. The findings from this study would guide policymakers on which factors to concentrate in the course of providing convenient, acceptable and effective online learning platforms especially in the context of distance education and even at the mainstream.

Essential variables in studying e-learning have been readily pointed out in literature by scholars from the areas of information systems and psychology (Sun et al., 2008). Among the variables, the “technology acceptance model” (TAM) of Ajzen and Fishbein (1977), Davis (1989), and Davis et al. (1989), in addition to the expectation and confirmation theory (ECT) credited to and Oliver (1980), Bhattacherjee (2001), Lin et al. (2005), and Wu et al. (2006) have partly contributed to understanding the success of e-learning. Furthermore, Al-adwan, Albelbisi, Hujran, Al-rahmi and Alkhalifah (2021), Opoku et al. (2020), and Pérez-Pérez et al. (2020) indicate that the information systems success model (ISSM) of DeLone and McLean (2003) offer a relevant contribution to explaining the dynamics of people's satisfaction with e-learning.

We, therefore model, in a single framework, the drivers of e-Learning satisfaction and perceived performance among distance learning students in Ghana in line with the TAM, ISSM, and ECT. We reveal, through this study, that instructors stand in a better position (through timely response, positive attitude, and diversified assessments) to induce and ignite the satisfaction levels of distance learning students in the context of e-learning since their attitudes usually influence the decisions of distance students.

The remaining parts of the paper follow this outline. Section 2 covers related literature and hypotheses development; Section 3 entails the methods; Section 4 details the results; Section 5 entails a discussion of the main results; we provide theoretical and practical implications in Section 6 and conclude in Section 7.

## 2 Literature review

Referring to the extant empirical literature, we place emphasis on six essential factors: technology anxiety (TA), instructor dimension (ID), course quality (CQ), technology quality (TQ), ease of use (EU), and perceived usefulness (PU). These factors are well-grounded in theories such as the TAM, the ISSM, and the ECT. The study maintains Sun et al.'s (2008) classification where TA is classified under learner dimension (LND), ID is under instructor dimension (IND), CQ is captured under course dimension (CSD), TQ under technology dimension (TGD), and EU and PU under design dimension (DSD).

### 2.1 Learner dimension (LND)

As confirmed by Arbaugh (2002), Arbaugh and Duray (2002), Hong (2002), and Piccoli et al. (2001), the attitude possessed by learners represents an essential factor in their satisfaction with e-learning. LND is defined as the impression held by learners from their participation in e-learning tasks via the use of a computer. In e-learning, computers are principally used as facilitating tools and are indispensable for effective e-learning service delivery (Sun et al., 2008). Piccoli et al. (2001) reveal that the anxiety of learners towards computers or technology (TA) has a significant effect on the satisfaction derived from e-learning. The media tools used in e-learning are basically computers and, therefore, fears with the use of computers would limit learner satisfaction (Piccoli et al., 2001; Sun et al., 2008). Cattell and Scheier (1961) advanced that anxiety is the consequence of mental pressure and comprises “trait” and “state” anxiety. Trait anxiety is determined by Spielberger (1976) to be an unchanging and intrinsic individual characteristic, whiles state anxiety is determined to emanate from the outside environment.

The extant literature suggests that TA falls in the category of state anxiety (Heinssen et al., 1987; Raub, 1981; Sun et al., 2008). As explained by Barbeite and Weiss (2004), TA is sensitive distress of probable negative consequences such as damaging the tool (e-learning technology). A learner with high anxiety about technology experiences a low satisfaction in learning. It is important to distinguish learners' anxiety from learners’ attitudes. The attitude of learners is just their beliefs and how they feel about computers (Heinssen et al., 1987). Existing studies suggest that TA affects the behaviours and attitudes of individuals and thus, the connection between TA and its impact on learning cannot be overlooked (Igbaria, 1990; Sun et al., 2008). In this study, anxiety is defined as the extent to which learners are anxious in the use of technological tools in e-learning. Following this, the study hypothesises that:

H1a learner technology anxiety has a negative effect on perceived satisfaction.

H1b learner technology anxiety has a negative effect on perceived performance.

### 2.2 Instructor dimension (IND)

The IND of e-learning comprises three aspects namely response timeliness, attitude towards e-learning tools, and readiness of diversified assessments for learners. Empirical works suggest that when instructors offer timely responses to learners, the satisfaction of learners is significantly influenced, as supported by Arbaugh (2002), Thurmond et al. (2002), and Sun et al. (2008). The justification is that there may exist the tendency for learners to await the response of instructors before resuming studies so a quick response from instructors would facilitate the prompt resumption of studies (Sun et al., 2008) on the part of learners. It is noted by Soon et al. (2000) that untimely response to problems of learners has an indirect effect on learners' performance. It, therefore stands to reason that the capability of instructors for prompt response improves learners’ satisfaction and learning outcomes, which corroborates the works of Arbaugh and Duray (2002), Ryan et al. (1999), Thurmond et al. (2002), Sun et al. (2008), and Islam and Azad (2015).

In this study, the response timeliness of instructors is operationalised as whether learners perceive that a facilitator offers a timely response to their difficulties. The attitudes possessed by team members or leaders towards technology is held to affect peoples' perception, as posited by Fulk, Schmitz and Steinfield's (1990) social influence model of technology. Fulk (1993) suggests that people's behaviours, actions, and sensitive reactions are observed by other individuals in an attempt to create their synchronised patterns of conduct. Piccoli et al. (2001), Webster and Hackley (1997), and Sun et al. (2008) determined that because instructors are key actors in learning activities, their attitudes towards the technological equipment used in e-learning has a positive consequence on the e-learning system.

In order to properly define learners' behaviour towards distance learning (e-learning), Sun et al. (2008) suggest that instructor behaviour towards the system should be as well studied. The perception of learners on the attitude of their tutor towards e-learning defines the attitude of instructors towards e-learning. Sun et al. (2008) suggest that efficient feedback mechanisms are essential to learners. In line with this, Thurmond et al. (2002) add that diversity in assessment is a key driver of learners' satisfaction with e-learning. With a system of diversified assessments, e-learners come to the realisation that they are linked to their instructors and that their learning efforts are effectively evaluated (Sun et al., 2008) by instructors. This study, in line with this, maintains that with a more diversified assessment from e-learning, learners' satisfaction is enhanced through the feedback they get from the assessments. The availability of varying forms and methods of assessment defines learners’ perception of diversity in assessment.

H2a instructor factors (response timeliness, attitude, and diversity in assessment) positively affect perceived satisfaction.

H2b instructor factors (response timeliness, attitude, and diversity in assessment) positively affect perceived performance.

### 2.3 Course dimension

An antecedent of learners' satisfaction with e-learning is also defined in terms of a well-designed e-learning course(s) (Sun et al., 2008), characterised by quality (that is, course quality, “CQ”), as suggested by Piccoli et al. (2001). The cooperative or constructive model of learning was used by Leidner and Jarvenpaa (1995) to explain that learners are able to develop top-notch intellectual models and create abstract knowledge through “media presentations” and collaborative interactions. Virtual features of e-learning such as supervision of learning procedures, hypermedia presentation of study materials, and online collaborative discussion and brainstorming, facilitate the effective establishment of learning models by learners and inspire them towards the continuous Use of e-learning platforms and resources (Piccoli et al., 2001; Sun et al., 2008). Hence, e-learning CQ serves as an essential determinant of learners’ satisfaction with e-learning. Hypothesis 3 is stated as:

H3a quality e-learning courses have a positive effect on learners' perceived satisfaction.

H3b quality e-learning courses have a positive effect on learners' perceived performance.

### 2.4 Technology dimension

Moreover, we find from early studies such as Webster and Hackley (1997) and Piccoli et al. (2001) that technology quality (TQ) has a significant effect on the perceived satisfaction of e-learners. Sun et al. (2008) suggest that e-learning tools should bear user-friendly features like learning and committing to memory limited simple concepts and expressive keywords so that they are regarded as hustle free. Such tools would be willingly patronised by learners because they come with just limited barriers (Amoroso and Cheney, 1991, 2015). Hence, the higher quality and dependability of e-learning technology results in higher satisfaction among learners. TQ is defined as learners’ perception of technology (including electronic boards, earphones, microphones, to mention a few) employed in e-learning. We hypothesise that:

H4a quality technology has a positive effect on learners' perceived satisfaction.

H4b quality technology has a positive effect on learners' perceived performance.

### 2.5 Design dimension

Also, PU and EU are the main aspects of the design dimension of e-learning. The TAM predicts and evaluates the tendency for users to embrace technology. PU and EU are the main tenets of Davis' (1989) TAM. Through the works of Arbaugh (2000, 2002), Arbaugh and Duray (2002), Atkinson and Kydd (1997), and Wu et al. (2006), we find that the principles of the TAM, as propounded by Davis (1989) are significant drivers of learners’ satisfaction with e-learning. Under the TAM, the propensity for task improvement consequential to the implementation of a system is defined as PU whereas the perception held by users on the ease with which a system could be adopted is defined as EU. The extant literature suggests that EU and PU influence the attitudes of users toward an e-learning system and subsequently affect their beliefs and behaviours in the course of adopting the system (Sun et al., 2008). The application of the TAM to e-learning suggests that as learners possess a good perception about the “usefulness and ease of use” of e-learning platforms, they develop positive attitudes toward the system and this enhances the experiences they develop through learning. Consequently, they develop high satisfaction and this increases the propensity for future use of e-learning systems (Arbaugh, 2000, 2002; Sun et al., 2008). We hypothesise, in line with the design dimension, that:

H5a learner perception of EU of e-learning technology has a positive effect on learners' perceived satisfaction.

H5b learner perception of EU of e-learning technology has a positive effect on learners' perceived performance.

### 2.6 Perceived satisfaction and performance

An effective and widely recognised measure (Pérez-Pérez et al., 2020) of individuals’ happiness and contentment with the use of a technology or system (Al-Busaidi, 2013) is identified by Wu et al. (2010) as satisfaction. Alamri (2019) suggests that the interrelation between perceived satisfaction and performance could generate influential synergies through the learning experience users acquire. Shih, Muñoz and Sánchez (2006) establish that when learners experience high levels of satisfaction, improved learning outcomes are achieved for a number of reasons (Pérez-Pérez et al., 2020).

The first is that inspiration, learner success, and course/programme pass rates induce satisfaction (Naaj et al., 2012). Secondly, learner motivation is influenced by satisfaction, as indicated by Chute et al. (1999), and serves as an essential driver for learner success (American Psychological Association [APA], 1997). Finally, satisfaction is tied to learners’ performance such that a satisfied learner scores high marks/grades which also ensure better grade point average (GPA) or cumulative grade point average (CGPA); a satisfied learner is more likely to take on additional courses or programmes via e-learning (Naaj et al., 2012), and is likely to recommend e-learning to others (Pérez-Pérez et al., 2020) or would be okay if e-learning is maintained by his/her institution for future courses or programmes. Ifinedo, Pyke and Anwar (2018), and Pérez-Pérez et al. (2020) confirm the positive relationship between perceived satisfaction and e-learner performance. The study, therefore, hypothesises that:

H6 perceived satisfaction with e-learning positively affects learners’ performance.

**Figure 1:**
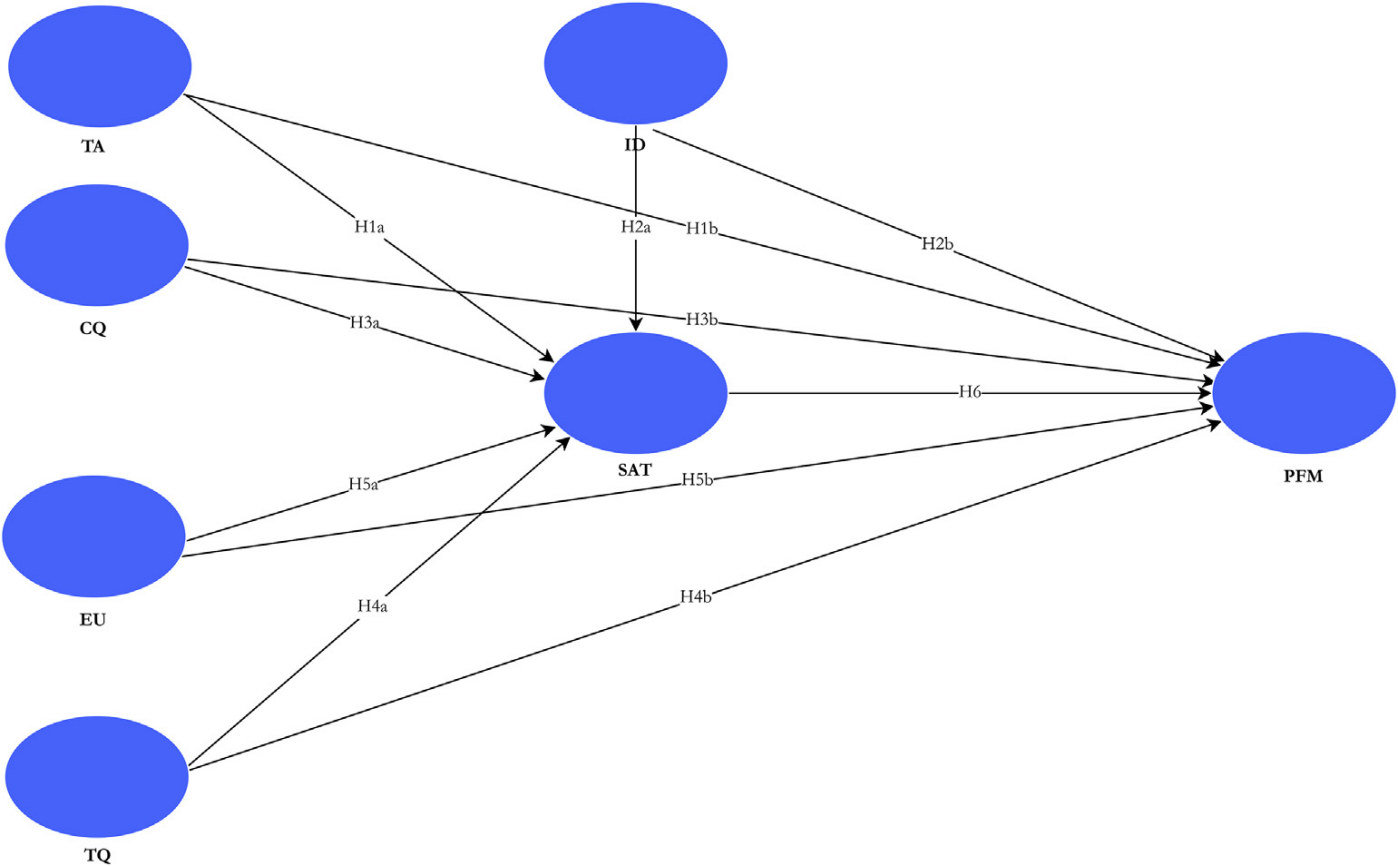
Conceptualised model of expected relationships. *Notes:* TA – technology anxiety; ID – instructor dimension; CQ – course quality; EU – ease of use; TQ – technology quality; SAT – satisfaction; PFM – performance.

Based on the concepts and empirical works reviewed, we conceptualise the study constructs in Figure 1.

As depicted in the conceptualised model in Figure 1, the study expected technology anxiety, course quality, technology quality, instructor factors, ease of use and expected usefulness to be significant drivers of learners’ perceived satisfaction and performance within an e-learning environment. These relationships, depicted by the path diagram, also represented the various hypotheses held by the study.

## 3 Methods

### 3.1 Research context and sample

We choose Ghana, a developing economy, as the study context for this research due to the following three reasons. First, the adoption of e-learning in Ghana was very low until the COVID-19 pandemic hit the country, forcing institutions to adopt e-learning across both the distance and regular levels. Second, the distance education system (DES) has gained considerable attention from the Ghanaian populace in recent times and the country seeks to widen the use of e-learning through the DES (Forson and Vuopala, 2019). Lastly, distance education offers a substantial contribution to socio-economic growth and for a developing economy like Ghana, a growth in the patronage for online arrangements vis-à-vis the DES calls for an investigation into what could drive learner satisfaction and performance using e-learning platforms; this would enable developing economies to focus on essential factors that facilitate effective e-learning across educational institutions.

The population of the study comprised all distance learning students across all tertiary institutions in Ghana. Specifically, students enrolled on distance learning programmes were all members of the study population. The accessible population, however, comprised distance learning students who had access to the internet and were registered on any social media platform. The size of the population, even though large, cannot be determined and thus, was unknown as at the conduct of the study. Therefore, the study's population size met the definition of an “unknown” but a large population as defined by Smith (2013).

A sample size of 388 was used in the study. The sample size was chosen to meet the minimum sample size of 385 required for a large but unknown population. The minimum sample size was determined through the formula propounded by Cochran (1977) and recently highlighted in Adam's (2020) study as

(1) $$n=\frac{Z_{\alpha /2}^{2}*p*q}{e^{2}},$$

where n is the size of the sample; and e represents the maximum tolerable error from estimation, which is set at 5% (i.e., 0.05), as recommended by Krejcie and Morgan (1970) and Adam (2020) when a data is deemed categorical; p signifies the population proportion, being the arithmetical chance of a success, which is set at 5% (i.e., 0.50) in line with the recommendation of Krejcie and Morgan (1970), to ensure a maximisation of the variance, resulting in generating a maximum sample size; q is the statistical chance of a failure and is equivalent to (1−p); and Z, which is expressed as (1−e), is the arithmetic parameter contingent on the level of confidence, which is specified as 95% (i.e., 0.95), and yields a value equivalent to 1.95, when read from the *t*-value table (Z-table.com, n.d.).

To have a fair overview of how the respondents were statistically distributed, the descriptive statistics of the respondents who participated in the study was generated. This covered all the 388 valid responses retrieved from the online survey. A summary of the demographic characteristics of respondents was presented in Table 1.

**Table 1:** Descriptive statistics of the sample.

Variable	*n*	%
Gender		
Male	221	56.96
Female	167	43.04

Age		
Below 20	14	3.61
20–30	285	73.45
31–40	76	19.59
41–50	11	2.84
Above 50	2	0.51

Marital Status		
Cohabiting	17	4.38
Divorced	4	1.03
Married	76	19.59
Never married	291	75.00
Widowed	0	0.00

Level of Study		
Diploma	45	11.60
Undergraduate	230	59.28
Research Postgraduate (M.Phil/M.Com/PhD)	41	10.56
Taught Postgraduate (MA/MBA/Med/PGDE)	72	18.56
Other	0	0.00

Affiliated Institution		
KNUST	27	6.96
Private University	18	4.64
Technical University	15	3.87
UCC	246	63.40
UDS	10	2.58
UEW	27	6.96
UG	8	2.05
Other	37	9.54

Field of Study		
Arts and Social Science	48	12.37
Business	197	50.77
Education	80	20.62
Mathematics and Sciences	54	13.92
Other	9	2.32

Skill in the use of e-learning Tools		
Novice	20	5.15
Intermediate	270	69.59
Expert	98	25.26

Skill in the use of e-learning Platform(s)		
Beginner	54	13.92
Intermediate	258	66.49
Advanced	76	19.59

*Notes:* N = 388. KNUST is Kwame Nkrumah University of Science and Technology; UCC is the University of Cape Coast; UDS is University for Development Studies; UEW is University of Education, Winneba; UG is the University of Ghana.

We observe from the descriptive summary in Table 1 that out of 388 respondents, the majority (*n* = 221) of the respondents who took part in the survey were males, representing 56.96%, with 167 females representing 43.04%. Of the 388 respondents surveyed, 76 (19.59%) were aged between 31 and 40 years. This indicated that a majority of the distance learning students in Ghana were males and were between the age bracket 20 and 40 years of age – and suggested that a youthful population of Ghana's economy are seeking education. Following this, it was not surprising that the vast majority (*n* = 291, 75%) of the respondents had never married as of the time they partook in the survey. Moreover, a few respondents (*n* = 72, 18.56%) indicated that they were enrolled on postgraduate research programmes as of the time of the conduct of the survey and an appreciable proportion (*n* = 41, 10.56%) were enrolled on postgraduate taught programmes. The majority (*n* = 230, 59.28%), however, were undergraduate students. This suggests that undergraduate distance education had received more attention over the years in Ghana. Diploma students numbered 45, representing 11.60% of the total respondents.

Concerning the skill of respondents with e-learning tools, a majority (*n* = 270, 69.59%) of them indicated that they were intermediate users of e-learning tools such as laptop or desktop computers, mobile phones, tablets, etc. Experts in the use of e-learning technology numbered 98, representing 25% of the 388 respondents who partook in the survey. Meanwhile, 20 (5.15%) respondents answered that they were new to e-learning technology. Notwithstanding, there was an indication that respondents could at least complete the survey with ease since over 90% were at least intermediate users of e-learning tools. Thus, the online survey posed little or no challenge for the respondents. Furthermore, 258 (66.49%) respondents indicated that they possessed intermediate skills with the e-learning platforms (e.g., LMS, Sakai, Zoom, Google Meet, etc.) available to them. Beginners and advanced e-learners numbered 54 (13.92%) and 76 (19.59%) respectively.

Overall, the demographic background of respondents suggests that most respondents were in the position to offer reliable answers to the questions involved in the survey. It was seen that even though only about 5% of respondents were new to e-learning tools, more than this proportion, approximately 14%, were beginners in the use of e-learning platforms. This suggests that policymakers and institutional heads for distance education should roll out measures that would offer some form of training to distance learning students especially in the area of e-learning. As Khadam et al. (2018) noted, the level of skills with e-learning platforms could influence learners’ satisfaction, and, to a large extent, their performance.

### 3.2 Instrument used and its validation

A closed-ended and self-administered questionnaire was used as the survey instrument. The survey instrument was randomly circulated on the social media handles of distance learning students. The question items were measured using a 7-point Likert scale, with ‘7’ representing the strongest agreement with a statement and ‘1’ representing the strongest disagreement. The instrument was hosted on google forms, allowing respondents the flexibility of participation. All the 388 valid responses were generated online and contained question items that served as a check for common method bias or variance (CMV). The instrument was arranged in seven google form pages, and all question items were “required” such that no respondent could skip any question once he/she agreed to participate in the survey. The relevant constructs (and their source(s)) that were employed in the study included technology anxiety of learners (TA) (Gattiker and Hlavka, 1992), instructor dimensions (ID) (Thurmond et al., 2002; Webster and Hackley, 1997), course quality (CQ) (Arbaugh, 2000), perceived ease of use (EU) of e-learning tools (Arbaugh, 2000), technology quality (TQ) (Amoroso and Cheney, 1991), satisfaction (SAT) (Arbaugh, 2000), and expected learner performance (PFM) (Damnjanovic et al., 2015; Hsieh and Cho, 2011). These constructs contained 4, 3, 2, 4, 4, 5, and 6 measurement items respectively. The question items on the demographic characteristics included the gender, age, marital status, the current level of study, affiliated institution, the field of study, the e-learning tools they are familiar with, respondents' rating of skill in the use of e-learning equipment, and their skill in the use of e-learning platforms. Further details to the instrument employed are provided in the supplementary material.

To meet ethical requirements, the instrument used for the study was vetted and approved by the representative of the supervising authority for ethical clearance of the University of Cape Coast, where the authors are engaged. The study complied with all regulations of the approving authority. Additionally, respondents were requested to complete the survey online only after they had agreed to do so. To ensure this, personal communications were made to the respondents before their participation and hence, made the process entirely voluntary. To achieve this task, the link to the survey was sent across several online platforms made up of Ghanaian distance learning students. The introductory part of the survey disclosed to respondents that the exercise was completely voluntary and they could exit at any stage. The survey lasted between 29 May 2021 and 25 June 2021. A total of 393 responses were generated from the survey over the period. However, the study excluded five (5) responses that were invalid. The data collection yielded 388 valid responses which were quite enough to be used for analysis using the SEM technique. According to Kline (2015), a minimum of 100 observations are required for estimating SEM whereas 200 data points are necessary for reliable estimates – this was met by this study since the valid responses numbered 388.

### 3.3 Data analysis

Prior to the regression analysis, several diagnostics were performed to ascertain the reliability and validity of path coefficients, the model fit, and common method bias. These are presented in detail as follows.

The Cronbach's alpha (CA) (Cronbach, 1951) and composite reliability (CR) statistics were employed in establishing the reliability of the measurement model. The study ensured that the CA and CR values for all the factors exceeded the required cut-off, 0.70, as proposed by Hair et al. (2010). Discriminant or divergent validity (DV) was used to confirm that constructs that are expected not to have any connection indeed did not relate to each other. To test for DV of the constructs, in this study, the squared-correlations of the unobserved variables were arranged in a correlation matrix and compared with their average variance explained (AVE), reported in Section 4. Hair et al. (2010) suggest that the DV of the constructs is confirmed only when the “squared-correlations below diagonal” does not exceed the AVE of all the unobserved variables.

Furthermore, the measurement errors that arise from methodical limitations are termed common method variance/bias (CMV). For example, the use of a typical 7-point Likert scale for all the questions in a survey may result in CMV. In this direction, Podsakoff et al. (2003) have suggested remedial actions to correct for CMV, for which there are specific merits and demerits for each approach. The study used the widely employed technique, which is the single factor test of Herman. By this approach, the study conducted an EFA where question items loaded on one latent factor. The AVE by the single factor was 0.48, which is less than the required cut-off – 50% (i.e., 0.50). Therefore, CMV was found not to be an issue in this research.

## 4 Results

### 4.1 Measurement model

The partial least squares structural equation modelling (PLS-SEM) estimator was employed to model the drivers of e-learning satisfaction and performance of distance learning students. The software employed in processing and analysing the data used in this study was Version 3.3.3 of Smart-PLS designed by Ringle et al. (2015). Regression results were generated using 5,000 bootstrap samples and the hypotheses were tested under a 5% level of significance. PLS-SEM contains a double examination: a description of the measurement model and an analysis of the structural model (Ringle et al., 2015). The structural model is used if the measurement model specification ensures that those constructs have adequate indicator loading, convergent validity, composite reliability, and discriminant validity. The evaluation of path coefficients and their significance is a part of structural model assessment. These assessments are provided as follows.

#### 4.1.1 Indicator factor loadings

Factor loadings represent the degree to which items from a given correlation matrix relate with a given principal component – loadings range between −1.0 and 1.0 where a higher value, in absolute terms, suggest a high correlation with a given factor (Pett et al., 2003). Table 2 contains the factor loadings for all indicators of this study.

**Table 2:** Indicator factor loadings, variance inflation factor, reliability and validity statistics.

	VIF	CQ	EU	ID	PFM	SAT	TA	TQ	CA	CR	AVE
Course Quality (CQ)									0.863	0.936	0.879
CQ1	2.362	0.946									
CQ2	2.362	0.929									
Ease of Use (EU)									0.955	0.967	0.881
EU1	4.451		0.924								
EU2	5.899		0.946								
EU3	5.205		0.936								
EU4	6.081		0.948								
Instructor Dimension (ID)									0.729	0.842	0.640
ID1	1.499			0.751							
ID2	1.572			0.801							
ID3	1.327			0.845							
Performance (PFM)									0.944	0.956	0.783
PFM1	3.737				0.896						
PFM2	2.626				0.827						
PFM3	5.944				0.938						
PFM4	5.637				0.928						
PFM5	4.221				0.908						
PFM6	2.478				0.802						
Satisfaction (SAT)									0.960	0.969	0.863
SAT1	4.956					0.928					
SAT2	5.612					0.933					
SAT3	5.343					0.934					
SAT4	4.933					0.922					
SAT5	5.034					0.928					
Technology Anxiety (TA)									0.856	0.901	0.696
TA1	1.655						0.770				
TA2	2.253						0.876				
TA3	2.992						0.904				
TA4	1.970						0.779				
Technology Quality (TQ)									0.904	0.933	0.778
TQ1	2.320							0.866			
TQ2	3.742							0.902			
TQ3	4.396							0.925			
TQ4	2.170							0.831			

*Notes:* CA is Cronbach Alpha, CR signifies composite reliability, and AVE is average variance extracted.

As indicated by Hair et al. (2010) and Hair et al. (2016), indicators are satisfactorily reliable if the items have loadings between 0.5 and 0.7. Following this, the reliability of the indicators used in the study was confirmed – the factor loadings exceeded 0.5 and ranged between a minimum and a maximum of 0.751 and 0.948 respectively, with significance at *p* < 0.001. Therefore, for the selected constructs, no indicators were removed except for the last three (SAT6, SAT7, and SAT8) indicators of satisfaction.

#### 4.1.2 Multicollinearity of indicators

In accordance with Fornell and Bookstein (2018), the study utilised the variance inflation factor (VIF) statistic to evaluate the collinearity in the indicators. The threshold for VIF is either argued as conservative, set at 5 (Hair et al., 2016), or high, when set at 10 (Alauddin and Nghiemb, 2010; Asthana, 2020; Gómez et al., 2016). For instance, Asthana (2020) states that multicollinearity is said to be present when the VIF is in excess of the rule of thumb 10. The indicator VIFs presented in Table 2 suggest that given the threshold of 10, all indicators did not suffer from multicollinearity.

#### 4.1.3 Reliability of constructs

An indicator is reliable if it is stable and consistent, and could aid replication in different contexts for the same outcomes. Principally, the Cronbach Alpha (CA) and the composite reliability (CR) are used to establish the reliability of latent variables and these were employed in this study. The resultant statistics of the two measures of reliability are presented in Table 2. The CA statistics ranged between 0.729 and 0.955 whereas the CR statistics ranged between 0.842 and 0.969. Thus, both measures of reliability yielded reliability statistics greater than the threshold of 0.70 suggested by Hair et al. (2014).

#### 4.1.4 Validity of constructs

For constructs to be valid in PLS-SEM, two tests are carried out – convergent and discriminant validity tests. Thus, convergent validity and discriminant validity confirm the validity of constructs when the PLS-SEM estimator is employed. Following this, the study carried out the two tests in confirming construct validity.

The extent to which multiple items ‘agree’ or ‘converge’ in their measure of the same constructs is known as convergent validity. The intuition is that there should exist a high covariance between two or more indicators of a single construct in order for them to be valid measurement indicators for the construct, as described by Bagozzi et al. (1991). To test for convergent validity, the study employed the approach suggested by Fornell and Larcker (1981), which suggests that convergent validity is confirmed when the AVE is at least 0.50. The convergent validity results on the basis of the AVE are presented in Table 1A in the Appendix. In this study, convergent validity was not an issue since all the AVE statistics were above the recommended cut-off of 0.50. The AVE statistics for the latent variables ranged between 0.640 and 0.881, as Table 1A presents.

Bagozzi et al. (1991) define discriminant validity as the extent to which measures of different constructs are unique. Thus, constructs should be different since they measure different concepts. Two or more constructs are distinct when they do not exhibit high correlations. The approaches to determining the discriminant validity among constructs include the Fornell and Larcker Criterion, cross-loadings, and the Heterotrait-Monotrait (HTMT) ratio. These measures were assessed to establish the discriminant validity for the constructs used in this study.

Suggested by Fornell and Larcker (1981), the Fornell and Larcker criterion holds that the square root of a construct's AVE should exceed its correlation with every other construct in a given model. The Fornell and Larcker criterion statistics are presented in Table 2A in the Appendix based on the square root of AVE and their correlations with other constructs. The results from the Fornell and Larcker criterion test (see Appendix) established the convergent validity among the constructs employed in the study since the square root of AVE for each construct proved greater than its co-movement with other constructs.

Another measure of discriminant validity is cross-loadings, which assesses whether an indicator for a construct loads much stronger on its parent construct relative to other constructs in a model (Wasko and Faraj, 2005). Thus, a factor should load better on its main construct than when tested on other constructs. The various loadings are presented in Table 3A in the Appendix. The cross-loadings from Table 3A (see Appendix) indicate that all indicators were better measures on their underlying constructs than when tested on other constructs in the study. Therefore, cross-loadings statistics also established the discriminant validity of the constructs employed in this study.

**Table 3:** Results of direct relationships.

	*β*	*SD*	*t*-stats	*p*-values	CI	
					2.5%	97.5%
H_1a_: TA -> SAT	-0.096	0.036	2.671	0.008	-0.165	-0.023
H_1b_: TA -> PFM	-0.008	0.036	0.230	0.818	-0.075	0.062
H_2a_: ID -> SAT	0.222	0.052	4.294	0.000	0.118	0.323
H_2b_: ID -> PFM	-0.098	0.046	2.123	0.034	-0.188	-0.011
H_3a_: CQ -> SAT	0.129	0.044	2.907	0.004	0.043	0.219
H_3b_: CQ -> PFM	0.042	0.041	1.017	0.309	-0.039	0.120
H_4a_: TQ -> SAT	0.235	0.062	3.826	0.000	0.116	0.359
H_4b_: TQ -> PFM	0.071	0.051	1.377	0.168	-0.024	0.175
H_5a_: EU -> SAT	0.323	0.067	4.802	0.000	0.196	0.459
H_5b_: EU -> PFM	0.197	0.052	3.797	0.000	0.096	0.299
H_6_: SAT -> PFM	0.638	0.048	13.396	0.000	0.539	0.727
	R^2^	Adjusted R^2^	Q^2^			
SAT	0.595	0.589	0.508			
PFM	0.635	0.629	0.489			

As a way of evaluating discriminant validity, the basis of the HTMT is the estimated correlations between multiple constructs in a study. With this approach, conclusions on the threshold are yet to be established. For example (Kline, 2011), recommended a cut-off point of 0.85 whereas (Teo et al., 2008) suggested a 0.90 cut-off point. This study presented the HTMT statistics in Table 4A in the Appendix. The HTMT results in Table 4A (see Appendix) suggested that none of the correlations exceeded the required threshold of either 0.85 or 0.90 as suggested respectively by Kline (2011) and Teo et al. (2008). Hence, the HTMT statistics also confirmed the discriminant validity of the constructs used in the study.

**Table 4:** Mediation analysis.

	Total Effect	*t*-stats	*p*-value	Direct Effect	*t*-stats	*p*-value		Indirect Effect	*t*-stats	*p*-value
CQ-PFM	0.124	2.446	0.000	0.042	1.017	0.309	CQ-SAT-PFM	0.082	2.829	0.005
EU-PFM	0.403	6.028	0.000	0.197	3.797	0.000	EU-SAT-PFM	0.206	4.588	0.000
ID-PFM	0.044	0.807	0.420	-0.098	2.123	0.034	ID-SAT-PFM	0.142	3.964	0.000
TA-PFM	-0.070	1.792	0.073	-0.008	0.230	0.818	TA-SAT-PFM	-0.061	2.637	0.008
TQ-PFM	0.221	3.398	0.001	0.071	1.377	0.168	TQ-SAT-PFM	0.150	3.730	0.000

### 4.2 Structural model

After evaluating the measurement model to ascertain its fitness, the study assessed the hypothesised relationships to substantiate the proposed hypotheses.

#### 4.2.1 Hypotheses testing

The various hypotheses are analysed based on the results of the direct relationships are summarised in Table 3. The reported results were based on 5,000 bootstrap samples, which also generate a two-tailed 95% confidence interval (CI). Following a CI, a significant relationship is characterised by a CI different from zero.

H1a learner technology anxiety has a negative effect on perceived satisfaction

H_1a_ examined whether technology (TA) anxiety negatively impacts the satisfaction (SAT) derived by e-learners from e-learning. The study found that TA has a significant negative influence on SAT (*β* = -0.096, *t* = 2.671, *p* < 0.01). Therefore, H_1a_ was maintained.

H1b learner technology anxiety has a negative effect on perceived performance

H_1b_ determined whether the technology anxiety of distance learning students engaged in e-learning influences their performance (PFM) or expected learning outcomes. The results suggested a negative but non-significant influence of TA on PFM (*β* = -0.008, *t* = 0.230, *p* = 0.818). The study finds insufficient evidence in support of H_1b_ and hence, fails to maintain H_1b_.

H2a instructor factors/dimensions (response timeliness, attitude, and diversity in assessment) positively affect perceived satisfaction

The aim of H_2a_ was to scrutinise the relationship between instructor dimensions (ID) or factors and the satisfaction derived from e-learning by distance learning students. The findings revealed that ID has a positive significant relationship with SAT (*β* = 0.222, *t* = 4.294, *p* < 0.001). Since the study found enough evidence to support that ID significantly influences SAT, H_2a_ was maintained.

H2b instructor factors (response timeliness, attitude, and diversity in assessment) positively affect perceived performance.

H_2b_ assessed whether instructor factors had a significant positive relationship with the expected learning performance from the perspective of distance learning students engaged in e-learning. The study found rather an indirect and significant effect of ID on PFM (*β* = -0.098, *t* = 2.123, *p* < 0.05). This result proves counter-intuitive. Subsequently, H_2b_ was not supported.

H3a quality e-learning courses have a positive effect on learners' perceived satisfaction

H_3a_ assessed the significance of the relationship between the quality of e-learning course(s) (CQ) and satisfaction of distance learning students enrolled in distance programmes. The results suggest that there exists a direct and significant effect of CQ on SAT (*β* = 0.129, *t* = 2.907, *p* < 0.01). With enough evidence gathered in support of this hypothesis, the study fails to reject H_3a_.

H3b quality e-learning courses have a positive effect on learners' perceived performance

H_3b_ examined whether the connection between course quality and learning outcomes was significant among distance learning students enrolled on e-learning systems. From the findings, there exist no significant influence of CQ and learning outcomes of distance education learners (*β* = 0.042, *t* = 1.017, *p* = 0.309). Thus, the evidence found was insufficient to support the claim that CQ would significantly influence PFM. Hence, H_3b_ was unsupported.

H4a quality technology has a positive effect on learners' perceived satisfaction

H_4a_ investigated whether the quality of e-learning technology (TQ) significantly affects the perceived satisfaction of distance learning students. The findings of the study revealed a positive and significant relationship exist between TQ and SAT (*β* = 0.235, *t* = 3.826, *p* < 0.001). having gained sufficient support for the hypothesis, the study failed to reject H_4a_.

H4b quality technology has a positive effect on learners' perceived performance

H_4b_ evaluated the significance of the relationship between the quality of e-learning technology and expected learning outcomes. The results generated indicate that the positive relationship that exists between TQ and PFM was statistically non-significant since the evidence found was insufficient (*β* = 0.071, *t* = 1.377, *p* = 0.168). Consequently, the study failed to maintain H_4b_.

H5a learner perception of EU of e-learning technology has a positive effect on learners' perceived satisfaction

H_5a_ assessed the significance of the relationship between ease of use (EU)of e-learning technologies and the level of satisfaction perceived by distance learning students who are engaged in e-learning. The study found a positive and statistically significant influence of EU on SAT (*β* = 0.323, *t* = 4.802, *p* < 0.001). With sufficient evidence in support of this hypothesis, the study fails to reject H_5a_.

H5b learner perception of EU of e-learning technology has a positive effect on learners' perceived performance

H_5b_ examined whether the ease with which e-learning technology significantly influences the performance of distance education students enrolled on e-learning programmes. The study found that EU significantly affects PFM (*β* = 0.197, *t* = 3.797, *p* < 0.001). Having generated sufficient evidence for this hypothesis, H_5b_ was supported.

H6 perceived satisfaction with e-learning positively affects learners' performance

**Figure 2:**
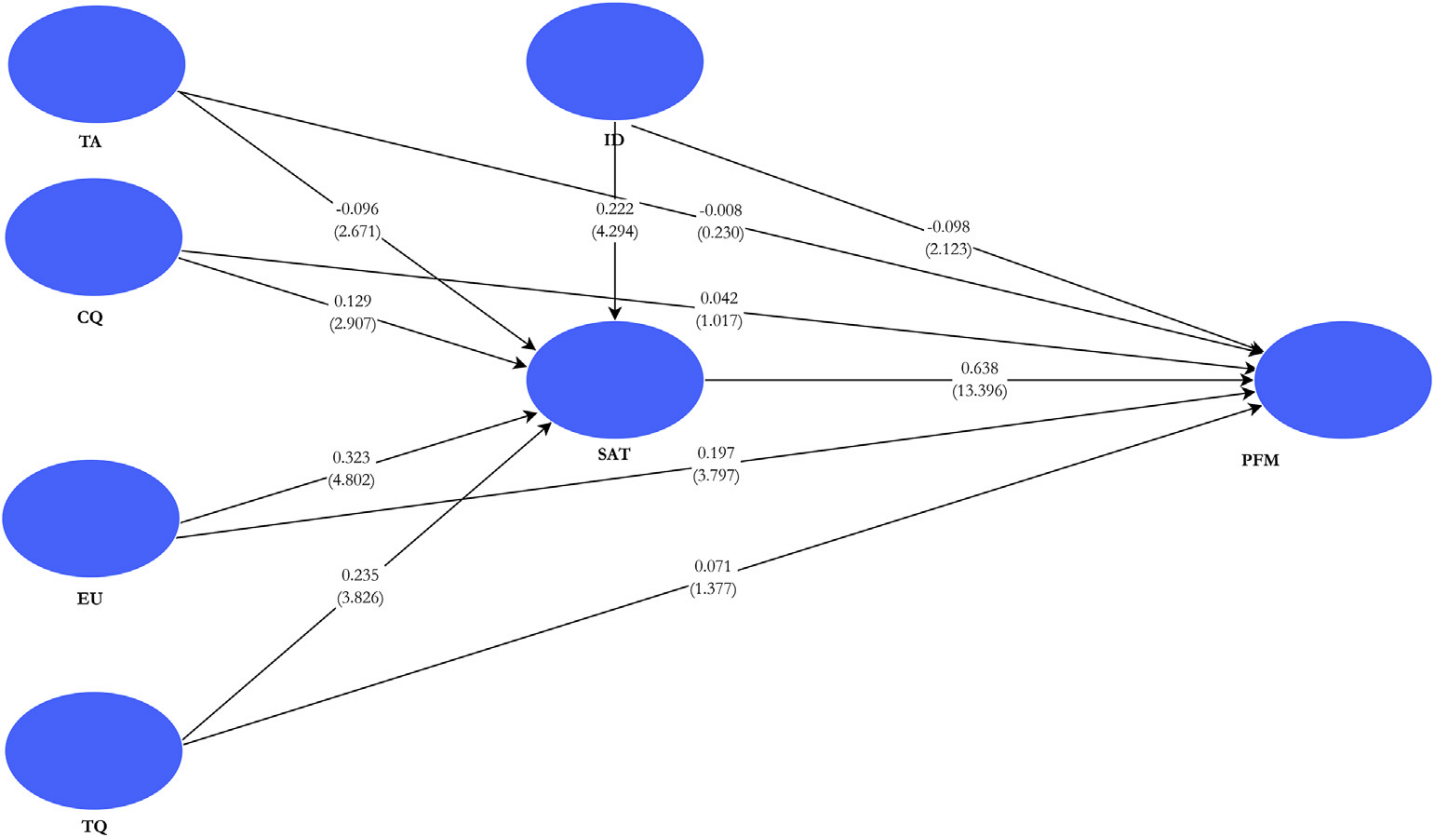
Structural model, path coefficients, and t-statistics. *Notes:* path coefficients in regular texts; *t*-statistics in parenthesis; R-squared values are in white fonts. TA – technology anxiety; ID – instructor dimension; CQ – course quality; EU – ease of use; TQ – technology quality; SAT – satisfaction; PFM – performance.

H_6_ investigated whether the satisfaction (SAT) perceived by e-learners substantially translates into performance (PFM) or learning outcomes among distance learning students. The results from the study suggest that SAT significantly improves PFM (*β* = 0.638, *t* = 13.396, *p* < 0.001). The results provide enough support for the acceptance of this hypothesis. Hence, H_6_ was maintained. The structural model with t-statistics was graphically represented in Figure 2.

#### 4.2.2 Analysis of mediation

From the modelled structure, it was deduced that the drivers of SAT influence PFM through SAT. Thus, SAT explains the distinct relationships between the drivers of SAT and expected performance. Without satisfaction, TA, ID, CQ, TQ, and EU may not fully explain PFM and hence, warrants an investigation into the nature of such indirect relationships. Following this, a mediation analysis was carried out and the results are summarised in Table 4. On mediation analysis, Zhao et al. (2010) offer a guide in interpreting results. According to them, aside from “no mediation,” mediation could be partial or complete. Partial mediation is one in which both the direct and indirect effects are significant; a complete mediation occurs when the indirect effect is significant but the direct effect proves non-significant.

From the results in Table 4, the mediation analysis could be summarised as the mediating role of SAT on the relationship between (1) CQ and PFM (2) EU and PFM, (3) ID and PFM, (4) TA and PFM, and (5) TQ and PFM. Concerning (1), the results revealed that the total effect of CQ on PFM was significant (*β* = 0.124, *t* = 2.446, *p* < 0.001). When the mediating variable (MV) was introduced, the effect of CQ on PFM was non-significant (*β* = 0.042, *t* = 1.017, *p* = 0.309). The indirect influence of CQ on PFM through SAT proved significant (*β* = 0.082, *t* = 2.829, *p* < 0.01). This suggested that the relationship between CQ and PFM is partially mediated by SAT.

Concerning (2), the results revealed that the total effect of EU on PFM was significant (*β* = 0.403, *t* = 6.028, *p* < 0.001). When the mediating variable (MV) was introduced, the effect of EU on PFM was still significant (*β* = 0.197, *t* = 3.797, *p* < 0.001). The indirect influence of EU on PFM through SAT proved significant (*β* = 0.206, *t* = 4.588, *p* < 0.001). This suggested that the relationship between EU and PFM is partially mediated by SAT.

With (3), the results revealed that the total effect of ID on PFM was non-significant (*β* = 0.044, *t* = 0.807, *p* = 0.420). When the MV was introduced, the effect of ID on PFM was still significant (*β* = -0.098, *t* = 2.123, *p* < 0.05). The indirect influence of ID on PFM through SAT proved significant (*β* = 0.142, *t* = 3.964, *p* < 0.001). This suggested that the relationship between ID and PFM is completely mediated by SAT.

In relation to (4), the results revealed that the total effect of TA on PFM was non-significant (*β* = -0.070, *t* = 1.792, *p* = 0.073). When the MV was introduced, the effect of TA on PFM non-significant (*β* = -0.008, *t* = 0.230, *p* = 0.818). The indirect influence of TA on PFM through SAT proved significant (*β* = -0.061, *t* = 2.637, *p* < 0.001). This suggested that the relationship between TA and PFM is completely mediated by SAT.

Concerning (5), the results revealed that the total effect of TQ on PFM was significant (*β* = 0.221, *t* = 3.398, *p* < 0.01). When the MV, the effect of TQ on PFM was non-significant (*β* = 0.071, *t* = 1.377, *p* = 0.168). The indirect influence of TQ on PFM through SAT proved significant (*β* = 0.150, *t* = 3.730, *p* < 0.001). This suggested that the relationship between TQ and PFM is partially mediated by SAT.

#### 4.2.3 Summary of hypotheses and implied mediating relationships

The study presented a summary of hypotheses and implied mediations in Table 5.

**Table 5:** Summary of hypotheses and implied mediations.

Hypothesis	*β*	*p*-value	Comment/Decision
H_1a_: TA -> SAT	-0.096	0.008	Maintained
H_1b_: TA -> PFM	-0.008	0.818	Unconfirmed
H_2a_: ID -> SAT	0.222	0.000	Maintained
H_2b_: ID -> PFM	-0.098	0.034	Unconfirmed
H_3a_: CQ -> SAT	0.129	0.004	Maintained
H_3b_: CQ -> PFM	0.042	0.309	Unconfirmed
H_4a_: TQ -> SAT	0.235	0.000	Maintained
H_4b_: TQ -> PFM	0.071	0.168	Unconfirmed
H_5a_: EU -> SAT	0.323	0.000	Maintained
H_5b_: EU -> PFM	0.197	0.000	Maintained
H_6_: SAT -> PFM	0.638	0.000	Maintained
Implied Mediations			
CQ-SAT-PFM			Partial mediation
EU-SAT-PFM			Partial mediation
ID-SAT-PFM			Complete mediation
TA-SAT-PFM			Complete mediation
TQ-SAT-PFM			Partial mediation

The summary in Table 5 suggested that seven (7) out of 11 hypotheses were maintained. Also, the study found evidence for 3 partial and 2 complete mediations, implied from the relationships between the drivers of e-learning satisfaction and performance.

## 5 Discussion

The purpose of this study is to analyse the drivers of e-learning satisfaction and performance among distance learning students in Ghana. The results of the study suggest that the technology anxiety of distance learners (TA) has an impact on the satisfaction perceived by distance learning students. The intuition is that the more a distance student fears or possesses attitudes against the use of e-learning tools such as computers and laptops, mobile phones, tablets, etc., the less satisfaction such a student holds for online learning. This finding is supported by Sun et al.'s (2008) observation that the way individuals feel about computers determines their attitudes toward online studies. However, the relationship between TA was found insignificant. This could be justified from the direction that the mere fact that some individuals may dislike the use of computers does not mean that they cannot learn through other means and, hence, the fear of computers, although could reduce motivation for online studies, would not necessarily reduce a person's performance.

Instructor factors were found to be significant predictors of distance e-learners’ satisfaction such that distance students are satisfied when they receive a timely response from instructors, when instructors themselves love to use the e-learning platform, and when there are a variety of assessments for use by the instructor. For instance, Bervell and Umar (2020) suggested that instructor anxiety towards e-learning impacts the adoption of e-learning and where adopted, impact the satisfaction of e-learners. Also, there exists a high tendency for distance learning students to wait on the response of their online course facilitators or instructors before resuming studies and, hence, a quick response from facilitators would allow distance students to quickly resume studies, as suggested by Islam and Azad (2015). In addition, as Thurmond et al. (2002) and Alamri (2019) revealed, the availability of diversified assessment makes distance e-learners come to the realisation that they are linked to their facilitators and that their learning efforts are effectively evaluated (Sun et al., 2008). Therefore, distance learning students tend to have increased satisfaction towards e-learning when instructors are offering a prompt response, positive attitude to e-learning platforms, and with diversified assessments.

On the contrary, instructor factors were found to negatively relate to the expected learning outcomes of distance learning students in Ghana. This result seems counter-intuitive, but from one angle, it could be argued that given that Ghanaian students are not so frequent with the use of more interactive technological appliances, instructors may offer their best concerning the online system in the areas of positive attitudes toward the system, quick response to students, and diversified assessments. Yet, students deprived of e-learning abilities may fail to appreciate the course/programme and, hence, could negatively impact their performance. This result could be justified further from the angle of familiarity and ease of use of e-learning systems. In a frontier economy like Ghana, advanced technological systems are less frequent and unconventional so with a sudden introduction of an online system, students may fail to appreciate from the onset and this would most likely result in poor learning outcomes (Farooq et al., 2020, 2021). Notwithstanding, the role of individual differences in the perception, attitude, and use of e-learning systems cannot go unnoticed when the discourse about e-learning and its acceptance is at play (Khadam et al., 2018).

Furthermore, the quality of e-learning course(s) (CQ) was found to significantly influence the satisfaction of distance learning students enrolled on e-learning programmes – meanwhile, the relationship between CQ and expected learning outcomes was statistically non-significant, suggesting that the extent to which distance learning students envisage the course(s) offered on online portals as quality determines how they feel about the course(s). Notably, the findings suggest a significant relationship between technology quality (TQ) and satisfaction but not performance. Following the information systems success model, TQ and information/course quality (CQ) help users to appreciate the use of an information system, as put forth by Seddon (1997), Hsieh and Cho (2011), Alamri (2019), Al-adwan et al., 2021, Opoku et al. (2020); Pérez-Pérez et al. (2020), etc. Thus, to a large extent, the study's findings corroborate the ISSM. Our findings do not commensurate with those of Damnjanovic et al. (2015) who found no significant relationship between information and system quality on learner satisfaction with e-learning. It is essential to note that CQ and TQ do not automatically result in improved satisfaction of distance learning students engaged in e-learning. This could be explained as such because a better system needs an effective operator or implementor and so distance learning students are required to make good use of quality courses and quality e-learning tools once they appreciate or are satisfied with them.

In addition, we found that ease of use (EU) of e-learning platforms significantly predicts both the satisfaction and learning outcomes of distance learning students. This result reinforces the technology acceptance model (TAM), as confirmed by Al-adwan et al., 2021, Farooq et al. (2020), Escobar-Rodriguez and Monge-Lozano (2012), Islam and Azad (2015), Alamri (2019), and Pérez-Pérez et al. (2020). Thus, with EU, when distance learning students perceive that their usage of a specific system would require little or no effort, they develop a high satisfaction with the system and tend to use it more frequently, thereby improving their performance. As a student develops acceptance for a given e-learning technology, security in usage also plays a key role. It is expected that a highly secured system would induce usage (Farooq et al., 2020). Furthermore, a significant effect of satisfaction on performance or learning outcomes was found among distance learning students. This confirmed the mediating effect of satisfaction on the relationship between the satisfaction drivers and the performance of e-learners at the distance level. This finding substantiated the conclusions of Islam and Azad (2015) and Pérez-Pérez et al. (2020) which suggested that the main predictor of learning outcomes in an e-learning context is satisfaction.

Consequently, within the context of this study, the substantial drivers of e-learning satisfaction and performance among distance learning students in Ghana include technology anxiety, instructor factors, course quality, technology quality, and ease of use. Satisfaction was found to mediate the relationships between drivers of satisfaction and learning outcomes of distance learning students in Ghana.

## 6 Theoretical and practical implications

Aside from corroborating theoretical models such as the technology acceptance model (TAM) and the information systems success model (ISSM), our findings make advancements to these theoretical frameworks. For instance, under the TAM, ease of use of an e-learning platform would connote that high satisfaction with the system would be developed by learners when they perceive that their usage of a specific system would require little or no effort. This makes students develop and use the system more frequently. It stands to reason, therefore, that through frequent use of the system, learner performance is enhanced; they would gain skills to help solve complicated problems in the future. Hence, we reveal that the TAM of Davis (1989) does not only induce satisfaction levels of e-learners, as Pérez-Pérez et al. (2020) and others confirm, but it also improves their learning outcomes. Thus, an effective e-learning system would help future learners and so policymakers should consider the ease of use of the e-learning systems they introduce.

As regards the ISSM, our findings also provide an advancement. We find a significant relationship between technology quality (TQ) and satisfaction but not performance. Impliedly, TQ does not always lead to increased satisfaction among distance learning students who participate in e-learning for the reason that without an effective implementor, a quality system does not yield expected results. Corollary to this, distance learning students are expected to make excellent use of quality courses and quality e-learning tools once they appreciate or are happy with them. Excellent use of the system would be achieved when students have the requisite know-how vis-à-vis the use of the system. Notwithstanding, aside from demographic and experience or skill factors (Khadam et al., 2018), Farooq et al. (2020) advocate that the security of the system also predicts users’ satisfaction and must be considered by the system developers and/or implementors.

Notably, a holistic success model that encapsulates a set of interrelated constructs are essential to effectively explain the dynamics of e-learning in the contemporary era, as Eom (2021) explicates. We, therefore, advocate that for the ISSM to be complete, the expectation-confirmation theory (ECT) of Oliver (1980) must be integrated. According to the ECT, client pleasure is essential for establishing and maintaining customer loyalty in the long run (Bhattacherjee, 2001). Consequently, e-learners must be satisfied at their first introduction to the system, which could be done through the conduct of intensive training and guide for e-learners regardless of their technological background. It is through this training and first-hand experience that learners would have pleasure in the system. In comparison to industrialised countries, developing economies, notably in Africa, are crawling (Tagoe, 2012; Palvia et al., 2018) in their adoption of e-learning and, hence, the approach towards adoption and/or implementation on a large scale needs to be quite tailored to the cultures of the users in frontier economies (Farooq et al., 2020).

Corollary to our findings and the theoretical advancements they offer, we submit that technologically anxious distance learning students have lower motivation for e-learning because their attitudes toward technology predict their response to e-learning systems. Instructors stand in a better position – through timely response, positive attitude, and diversified assessments – to induce and ignite the satisfaction levels of distance learning students in the context of e-learning since their attitudes usually influence the decisions of distance students. Moreover, user-friendly e-learning platforms would cause distance learning students to appreciate e-learning – and this would, in turn, boost their performance (Farooq et al., 2020; Farooq et al., 2021; Khadam et al., 2018). Additionally, we expound that without learner satisfaction, distance learning students would not perform as expected in the context of e-learning. Thus, satisfaction is essential to ensuring that instructor factors and technology anxiety of distance learning students significantly induce their performance in an e-learning environment. Notwithstanding, as underscored by Khadam et al. (2018), we reiterate that regulators must account for individual differences when rolling out e-learning policies. Thus, regardless of the ease of use, perceived usefulness, instructor factors, quality of the course and/or technology, background issues such as gender, age, and skill level could all influence satisfaction and/or performance of e-learners.

We recommend that policymakers and educational institutions develop regular training for both tutors/instructors/facilitators and students so that the basic skill required for operating technological equipment and e-learning platforms could be imparted to all distance learning students within their reach. This would enable both instructors and students to appreciate the e-learning system for improved performance. More importantly, the channel through which the determining factors of e-learning satisfaction affect the performance of e-learners should be of great concern to governments and educational policymakers in developing economies in their journey to catching up with their counterparts in the advanced world. Furthermore, user-friendly systems should be employed rather than complex e-learning mechanisms. Following the technology acceptance model, students and instructors would only use a system when they perceive it as useful and requires little effort. Regular surveys should be conducted to assess the usefulness of an e-learning platform from the perspective of both learners and tutors as relevant feedback could be retrieved to always make the process a better one for users.

## 7 Conclusions, limitations, and future research

E-learning is envisaged to be employed as a mainstream teaching and learning mechanism by 2025. Developing economies – who aim to catch up with their counterparts in the advanced economies – are now picking up with their adoption and/or implementation of e-learning systems given the relevance of the system in fostering globalisation and integration among regions. There is the need, therefore, for governments and policymakers across developing economies to know what factors to consider in devising effective adoption and/or implementation policies. This study investigated the essential drivers of e-learning satisfaction and performance among distance learning students in the Ghanaian context. The study was guided by the technology acceptance model, the information systems success model, and the expectation-confirmatory theory. Data was gathered using an online survey between 29 May 2021 and 25 June 2021, generating 388 valid responses from distance education students spread across Ghana and had access to the link to the survey instrument. Under the PLS-SEM paradigm, we employed Version 3.3.3 of Smart-PLS to process and analyse the data gathered from the online survey. Assessments of the measurement and structural models were carried out.

In response to the study's research questions, we find that firstly, the factors that drive the perceived learning satisfaction of distance students include: technology anxiety, instructor factors, course quality, technology quality, and ease of use. Secondly, our results suggest that satisfaction is a significant predictor of learning outcomes for distance students in an e-learning environment. Lastly, satisfaction was found to mediate the relationships between drivers of satisfaction and learning outcomes of distance learning students in Ghana. More importantly, satisfaction was found a complete mediator of the relationship between each of instructor factors and performance of distance learning students, and technology anxiety of learners and performance of distance learning students in an e-learning environment. The theoretical and practical implications of the study offer essential policy directions for frontier economies aiming to catch up with their developed counterparts through the adoption or implementation of e-learning.

While our research contains some intriguing discoveries and insights, it also has several flaws that should be noted. First, although our results may be limited in their generalisability outside the country and platform contexts in which they were gathered, they are based on a rather large sample compared to certain previously published research. Second, the results provide important information on users of e-learning platforms from a developing economy relative to most previous studies that derived their analyses from data scraped from advanced economies. Nonetheless, having gathered data from multiple institutions across all regions in the country might improve the results’ generalisability and shed light on the possible influence of satisfaction determining factors on the performance of students across economies. Third, although other studies consider several dimensions of e-learning satisfaction, our study did not involve all of such dimensions. Notwithstanding, we employed dimensions that are essential to serving the purpose of the study and these dimensions are backed by the extant literature. Lastly, although statistical and software flaws might occur in data processing and analysis, we addressed common method bias issues and checked for convergent and discriminant validity to ensure that these issues did not exist in our data.

Future research could incorporate other dimensions of e-learning – based on a holistic systems success model that integrates a set of interconnected constructs, as Eom (2021) reports – to determine their influence on e-learning satisfaction and performance in the contemporary era. The study could further be applied in other contexts to assess plausible differences or corroborate our findings. Future studies could additionally focus on instructors or facilitators of e-learning systems rather than students.

## Declarations

### Author contribution statement

Ahmed Bossman; Samuel Kwaku Agyei: Contributed reagents, materials, analysis tools or data; Wrote the paper.

### Funding statement

This research did not receive any specific grant from funding agencies in the public, commercial, or not-for-profit sectors.

### Data availability statement

Data included in article/supplementary material/referenced in article.

### Declaration of interests statement

The authors declare no conflict of interest.

### Additional information

No additional information is available for this paper.

## Figures and Tables

**Table 1A:** Average variance extracted

Latent Variable	Average Variance Extracted (AVE)
CQ	0.879
EU	0.881
ID	0.640
PFM	0.783
SAT	0.863
TA	0.696
TQ	0.778

*Notes:* TA – technology anxiety; ID – instructor dimension; CQ – course quality; EU – ease of use; TQ – technology quality; SAT – satisfaction; PFM – performance.

**Table 2A:** Square-root of AVE and indicator correlations

Latent Variable	CQ	EU	ID	PFM	SAT	TA	TQ
CQ	**0.938**						
EU	0.386	**0.939**					
ID	0.534	0.595	**0.800**				
PFM	0.384	0.643	0.478	**0.885**			
SAT	0.462	0.687	0.622	0.779	**0.929**		
TA	-0.099	-0.236	-0.117	-0.229	-0.261	**0.834**	
TQ	0.340	0.679	0.541	0.575	0.639	-0.212	**0.882**

*Notes:* figures in bold represent the square root of AVE for the given construct. TA – technology anxiety; ID – instructor dimension; CQ – course quality; EU – ease of use; TQ – technology quality; SAT – satisfaction; PFM – performance.

**Table 3A:** Cross loadings

	CQ	EU	ID	PFM	SAT	TA	TQ
CQ1	**0.946**	0.357	0.526	0.378	0.464	-0.111	0.319
CQ2	**0.929**	0.368	0.472	0.341	0.397	-0.072	0.318
EU1	0.348	**0.924**	0.585	0.602	0.645	-0.159	0.639
EU2	0.348	**0.946**	0.543	0.578	0.624	-0.252	0.641
EU3	0.405	**0.936**	0.550	0.618	0.637	-0.219	0.608
EU4	0.348	**0.948**	0.556	0.615	0.672	-0.255	0.659
ID1	0.524	0.358	**0.751**	0.276	0.379	-0.095	0.356
ID2	0.507	0.407	**0.801**	0.305	0.463	-0.038	0.383
ID3	0.322	0.604	**0.845**	0.507	0.602	-0.134	0.523
PFM1	0.367	0.541	0.427	**0.896**	0.685	-0.189	0.513
PFM2	0.242	0.632	0.491	**0.827**	0.707	-0.204	0.574
PFM3	0.378	0.600	0.425	**0.938**	0.731	-0.204	0.536
PFM4	0.385	0.532	0.415	**0.928**	0.681	-0.165	0.468
PFM5	0.312	0.602	0.433	**0.908**	0.703	-0.193	0.526
PFM6	0.364	0.490	0.330	**0.802**	0.614	-0.264	0.418
SAT1	0.414	0.638	0.606	0.711	**0.928**	-0.248	0.605
SAT2	0.387	0.643	0.581	0.742	**0.933**	-0.240	0.611
SAT3	0.480	0.648	0.574	0.737	**0.934**	-0.244	0.594
SAT4	0.460	0.632	0.572	0.701	**0.922**	-0.222	0.570
SAT5	0.403	0.630	0.554	0.726	**0.928**	-0.259	0.586
TA1	-0.098	-0.156	-0.061	-0.181	-0.177	**0.770**	-0.186
TA2	-0.070	-0.245	-0.170	-0.223	-0.287	**0.876**	-0.206
TA3	-0.059	-0.224	-0.112	-0.197	-0.232	**0.904**	-0.173
TA4	-0.127	-0.129	0.008	-0.146	-0.131	**0.779**	-0.130
TQ1	0.366	0.576	0.463	0.560	0.550	-0.240	**0.866**
TQ2	0.260	0.638	0.537	0.518	0.594	-0.129	**0.902**
TQ3	0.285	0.638	0.485	0.508	0.587	-0.160	**0.925**
TQ4	0.288	0.536	0.418	0.436	0.519	-0.227	**0.831**

*Notes:* TA – technology anxiety; ID – instructor dimension; CQ – course quality; EU – ease of use; TQ – technology quality; SAT – satisfaction; PFM – performance.

**Table 4A:** Heterotrait-Monotrait ratio

Construct	CQ	EU	ID	PFM	SAT	TA	TQ
CQ							
EU	0.426						
ID	0.704	0.68					
PFM	0.427	0.675	0.542				
SAT	0.504	0.717	0.715	0.817			
TA	0.122	0.249	0.165	0.251	0.273		
TQ	0.385	0.729	0.642	0.618	0.685	0.238	

*Notes:* TA – technology anxiety; ID – instructor dimension; CQ – course quality; EU – ease of use; TQ – technology quality; SAT – satisfaction; PFM – performance.

## Appendix A Supplementary data

Drivers of e-Learning - Survey items Drivers of e-Learning - Survey items
