# Supplementary material for: Technology and instructor dimensions, e-learning satisfaction, and academic performance of distance students in Ghana
Source: Heliyon. 2022 Mar 31;8(4):e09200. doi: 10.1016/j.heliyon.2022.e09200 (PMC8987389; doi:10.1016/j.heliyon.2022.e09200)
Supplement: Drivers of e-Learning - Survey items [file mmc1.docx]

**Technology and Instructor Dimensions, e-Learning Satisfaction, and Academic Performance of Distance Students in Ghana**

**Survey Instrument**

| **Dimensions and Statements** | **Source(s)** |
| --- | --- |
| *Computer anxiety of learner* | Gattiker and Hlavka (1992) |
| 1. Working with a computer/e-learning tool would make me very nervous [R] |  |
| 1. I am bored by the use of e-learning technology [R] |  |
| 1. E-learning tools make me feel uncomfortable [R] |  |
| 1. Computers make me feel uneasy and confused [R] |  |
|  |  |
| *Instructor dimension* |  |
| 1. I received comments on assignments or examinations for the course(s) in a timely manner | Thurmond et al. (2010)  Webster and Hackley (2017) |
| 1. I think that instructors consider web-based learning more useful than the traditional classroom approach |  |
| 1. Taking a course via the Internet allows me to plan for the course more effectively |  |
|  |  |
| *Technology quality* |  |
| 1. I feel the information technology tools used in e-learning are very easy to use | Amoroso and Cheney (1991) |
| 1. I feel the information technology tools used in e-learning have many useful functions |  |
| 1. I feel the information technology tools used in e-learning are flexible |  |
| 1. I feel the information technology tools used in e-learning are easy to obtain |  |
|  |  |
| *Course quality* |  |
| 1. Courses conducted via the Internet have better quality compared to other means | Arbaugh (2000) |
| 1. The quality of courses taken online is largely unaffected by conducting it via the Internet |  |
|  |  |
| *Ease of use* |  |
| 1. It would be easy for me to become skilful at using web-based learning systems | Arbaugh (2000) |
| 1. Learning to operate web-based learning systems would be easy for me |  |
| 1. I would find it easy to get a web-based learning system to do what I want it to do |  |
| 1. I would find web-based learning systems easy to use |  |
|  |  |
| *Perceived satisfaction* |  |
| 1. I am satisfied with my decision to take this course via the Internet | Arbaugh (2000) |
| 1. If I had an opportunity to take another course via the Internet, I would gladly do so |  |
| 1. My choice to take this course via the Internet was a wise one |  |
| 1. I was very satisfied with the course via the online platform |  |
| 1. I will take as many courses via the Internet as I can |  |
| 1. I was disappointed with the way this course worked out [R] (dropped) |  |
| 1. If I had it to do over, I would not take this course via the Internet [R] (dropped) |  |
| 1. Conducting the course via the Internet made it more difficult than other courses I have taken (dropped) |  |
|  |  |
| *Performance/learning outcomes* |  |
| 1. E-learning improves my grade for the course | Self-developed, guided by Hsieh and Cho (2011)  Damnjanovic et al. (2015) |
| 1. Applying e-learning encourages me to continue learning on the Internet by myself |  |
| 1. I score high marks on assessments for courses held online |  |
| 1. My GPA/CGPA has improved as a result of e-learning |  |
| 1. I can attain the desired class I want through e-learning |  |
| 1. I would be okay if the institution goes ahead with e-learning for the remaining period of my programme/course of study |  |

Notes: [R] signify the question items that were coded in reverse; (dropped) indicate the questions items that were not used in the study for the particular construct; all question items were measured on a 7-point Likert scale with “1” representing “strongest disagreement” to a statement and “7” signifying “strongest agreement with a statement”
